# Unbiased Profiling Reveals Compartmentalization of Unconventional T-Cells Within the Intestinal Mucosa Irrespective of HIV Infection

**DOI:** 10.3389/fimmu.2020.579743

**Published:** 2020-09-30

**Authors:** Magalli Magnoumba, Alveera Singh, Paul Ogongo, Julia Roider, Osaretin Asowata, Michael Fehlings, Farina Karim, Thumbi Ndung'u, Frank Anderson, Alasdair Leslie, Henrik Kløverpris

**Affiliations:** ^1^Africa Health Research Institute (AHRI), University of KwaZulu-Natal (UKZN), Durban, South Africa; ^2^Institute of Primate Research, National Museums of Kenya, Nairobi, Kenya; ^3^Department of Infectious Diseases, Medizinische Klinik IV, Ludwig-Maximilians-University Munich, Munich, Germany; ^4^German Center for Infection Research (DZIF), Partner Site Munich, Munich, Germany; ^5^ImmunoScape Pte Ltd, Singapore, Singapore; ^6^HIV Pathogenesis Programme, Doris Duke Medical Research Institute, University of KwaZulu-Natal, Durban, South Africa; ^7^Division of Infection and Immunity, University College London (UCL), London, United Kingdom; ^8^Department of Surgery, Inkosi Albert Luthuli Hospital, Durban, South Africa; ^9^Department of Immunology and Microbiology, University of Copenhagen, Copenhagen, Denmark

**Keywords:** unconventional T-cells, MAIT cells, iNKT cells, intestinal mucosa, HIV infection, TCR repertoire, γδ T-cells, gut compartmentalization

## Abstract

The intestinal mucosa is enriched for unconventional T-cells, including mucosal associated invariant T-cells (MAIT), invariant natural killer T-cells (iNKT) and γδ T-cells. These cells are activated by bacterial metabolites, lipid antigens and cytokines, and are important for intestinal barrier integrity. The loss of gut homeostasis observed in HIV infection is central to disease pathogenesis, and studies have highlighted impairment of particular unconventional T-cell subsets within a specific gut compartment. However, although the small and large intestine are distinct niches, the overall impact of HIV on unconventional T-cells across the gut mucosal has not been well-studied. We hypothesized that compartment specific differences in the unconventional T-cell repertoire would exist between the small and large intestine, due to increasing bacterial loads and microbial diversity; and that the impact of HIV infection might differ depending on the compartment examined. We used mass cytometry, flow cytometry and unbiased T-cell receptor profiling to quantify unconventional T-cells in blood and tissue from the small (duodenum) and large (colon) intestine in HIV infected and uninfected participants undergoing examination for a range of intestinal conditions. Overall, we find distinct compartmentalisation of T-cells between blood, duodenum and colon, with iNKT cells significantly enriched in the duodenum and δ-1 expressing γδ T-cells in the colon. In addition, we observe greater clonal expansion of conventional TCRs in the duodenum, suggestive of stronger adaptive immunity in this compartment. Conversely, we find evidence of an expanded unconventional TCR repertoire in the colon, which contained far more overlapping “donor unrestricted” sequences than the duodenum. Twelve of these TCRs were highly “MAIT-like” and 3 were unique to the colon, suggesting an enrichment of donor unrestricted T-cells (DURTs) in this compartment. Unexpectedly, however, no significant impact of HIV infection on any of the unconventional T-cell subsets measured was observed in either mucosal site in terms of frequency or TCR repertoire. Further studies are required to investigate the importance of these unconventional T-cell subsets to intestinal homeostasis within the different gut compartments and determine if they are functionally impaired during HIV infection.

## Introduction

Unconventional T-cells play a unique role in the immune response to viral and microbial pathogens by combining the antigen specificity of adaptive immunity with the rapid responsiveness of the innate immune system (1). These cells include mucosal-associated invariant T (MAIT) cells and Type I natural killer T (iNKT) cells, which both express semi-invariant αβ T-cell antigen receptors (TCRs) and gamma delta T-cells, which use the γδ-TCR.

Both MAIT and iNKT cells belong to the group of ‘Donor Unrestricted T-Cells (DURTs) which, unlike conventional TCRs, are expressed in all individuals. This stems from the fact that, unlike conventional T-cells that recognize antigen presented by diverse MHC molecules, the antigen targets of DURTs are presented by non-polymorphic molecules that are ubiquitously expressed. MAIT cells recognize microbial riboflavin metabolites bound to the monomorphic MHC class I–like protein MR1 using a restricted TCR-alpha repertoire that is strongly biased toward a TRAV1-2/TRAJ33 pairing. The TCR-beta usage of MAIT cells also appears to be restricted, although this has been less well-characterized (2). As their name suggests, MAIT cells are enriched at mucosal sites, including the intestinal mucosa (3), and they are important for protection against a range of bacteria, viruses and yeast (4–6) by complementing the conventional innate and adaptive immune response (7). Likewise, iNKT cells recognize glycolipid antigens presented by the non-polymorphic MHC class I-like molecule CD1d, through a single TRAV10/TRAJ18 alpha TCR and a restricted TCR-beta usage (8). These cells also appear to be important in tissue, and have been implicated in ameliorating or exacerbating a variety of diseases and illnesses, ranging from autoimmunity to cancer (9). In humans, MAIT cells compromise around 1–10% of peripheral blood T-cells whereas iNKT cells represent only ~0.1% (10, 11).

Gamma-delta (γδ) T-cells also rely primarily on monomorphic, non-MHC molecules, for antigen recognition but, unlike DURTS, they do not express semi variant TCRs (12). Nonetheless, γδ T-cells are much less diverse than conventional αβ T-cells, in part because the great majority of these TCRs are from only 3 delta chains (TRDV1-3) (13, 14). Similar to DURTs, the distribution of γδ T-cells appears to be highly compartmentalized and although they only represent a small fraction of circulating and lymphoid T-cells, γδ T-cells form a substantial population in tissue, where they can outnumber conventional αβ T-cells (14). Human γδ T-cells expressing the Vδ1 chain are mostly found in mucosal tissues and regarded as important for intestinal barrier function, while Vδ2 chain γδ T-cells (preferentially paired to the Vγ9 chain) predominate in the peripheral blood and secondary lymphoid organs (15). Based on their effector properties, Vγ9Vδ2^+^ T lymphocytes are believed to play an important role in cellular immune responses against tumors (16) and infectious diseases (17, 18). Vδ3 are less well-studied, but also appear to be enriched at mucosal barriers and in the liver (19).

Data from both human and animal studies indicate that the primary function of unconventional T-cells is at the barrier sites with the exterior environment, such as skin, lung, the genital and the intestinal tracts (10, 14). The gut is a key site for HIV pathogenesis and is a source of massive viral replication during the early stages of infection, resulting in the profound depletion of mucosal effector CD4 T-cells, including T helper (Th) 17 and Th22 cells that are critical for gut integrity [reviewed in (20)]. Epithelial barrier disruption is associated with local and systemic inflammation and movement of bacterial products through the lamina propria into circulation (21).

HIV infection is thought to have a profound impact on MAIT and iNKT cells (11). Both subsets are depleted and functionally impaired in peripheral blood of HIV infected individuals, and are only partially restored by antiretroviral treatment (ART) (22–25). The depletion of MAIT and iNKT cells has also been reported in gut tissue, supporting the hypothesis that HIV driven depletion of these cells directly contributes to impairment of mucosal immunity and gut barrier breakdown in HIV infected individuals (24). Likewise, HIV infection is associated with perturbation of γδ T-cells, resulting in a significant expansion of Vδ1^+^ and contraction of Vδ2^+^ γδ T-cell populations in the peripheral blood, and in the colonic mucosa (26, 27). These changes occur during acute HIV-1 infection and persist into the chronic phase of infection despite effective antiretroviral therapy (28). Indeed, in a recent study, pro-inflammatory Vδ1^+^ cell frequency was found to correlate with levels of HIV RNA in intestinal tissue but not in plasma (29). Although these studies highlight the compartment specific activity of these unconventional T-cells and support a probable role in HIV pathogenesis, data on mucosal unconventional T-cell subsets in HIV infection remains scarce (11). Moreover, probably for practical reasons, these few studies have tended to focus on the large intestine and extrapolated their findings to the whole “gut.” The luminal microenvironment in the small intestine (e.g., duodenum) and colon, however, is inherently different, reflecting their specific functions and the distinct microbial communities they support (30). It is highly likely that this also impacts the T-cell repertoire of each compartment. However, the T-cell landscape of the different intestinal compartments is not well-understood, and studies addressing tissue-specific compartmentalization and the effect of HIV infection on unconventional T-cells are rare (11).

Here, we investigate the unconventional T-cell subsets present within the duodenum and the colon and test the hypothesis that this repertoire is highly compartmentalized across the gastrointestinal tract and differentially impacted by HIV infection. We apply 3 parallel approaches within duodenum, colon and blood compartments, (i) high dimensional mass cytometry to provide detailed phenotyping of the conventional DURTs and γδ T-cell populations; (ii) flow cytometry to perform direct comparisons between HIV infected and uninfected participants; and iii) using a novel approach, make use of the restricted TCR-alpha repertoire or DURTs and delta chain usage of γδ T-cells to quantify all 3 subsets by unbiased global TCR-sequencing (31).

## Methods

### Human Cohorts

To study immunity at the site of disease, we recruited 85 participants with mixed ethnical background undergoing endoscopies for clinical indications at Inkosi Albert Luthuli Central Hospitals in Durban, KwaZulu-Natal province in South Africa (GI cohort). The considerable variability between individuals prevented the clinical categorization of participants, but identified pathologies ranged from benign conditions to confirmed gastrointestinal malignancies (Table 1 and Supplementary Table 1 for individual participants' characteristics). Duodenal pinch biopsies (*n* = 30) (30) were obtained during gastroscopies or endoscopic retrograde cholangiopancreatographies (ERCPs), colon pinch biopsies (*n* = 23) during coloscopies. In 46/85 individuals, matched blood and tissue samples could be obtained. Additionally, PBMC samples of 26 HIV uninfected females with sub-Saharan Zulu/Xhosa ancestry from the Females Rising through Education Support and Health (FRESH) cohort (32) were included as healthy controls without underlying gastrointestinal diseases. All participants provided informed consent and each study was approved by the respective institutional review boards including the Biomedical Research Ethics Committee of the University of KwaZulu-Natal for all the studies.

**Table 1:** Clinical characteristics of *n* = 111 individuals used in study.

		**Non-GI HIV^**−**^ (*n =* 26)**	**GI HIV^**−**^ (*n =* 62)**	**GI HIV^**+**^ (*n =* 23)**
Age yrs median (IQR)		21 (19–22)	45 (36-59)	41 (33-51)
Gender	F/M	26/0	32/30	21/2
Ethnicity (%)	Black	26 (100)	26 (42)	21 (91)
	Colored	0 (0)	7 (11)	2 (9)
	Indian	0	22 (35)	0 (0)
	White	0	7 (11)	0 (0)
Compartment	Blood	26	22/62	10/23
	Blood+Tissue	0	35/62	11/23
	Tissue	0	5/62	2/23
Assay	CYTOF	0	7	0
	FACS	26	34	15
	TCR	0	21	8
Yrs median (IQR) from diag		NA	NA	7 (3-11)
Viral load (<20/>20/ND)		NA	NA	18/2/3

### Sample Preparation

#### PBMC Isolation

Blood was collected in BD vacutainers (sodium heparin, BD), PBMCs were isolated using Ficoll-Histopaque (Milli-pore Sigma) density gradient centrifugation and cryopreserved in freezing media (10% DMSO; 90% FCS) until when needed. All PBMC samples were frozen before use, except experiments for Mass Cytometry.

#### Processing of Tissue

Colon and duodenum were used as the different parts of the GI, removed as a biopsy and were collected in RPMI media (RPMI 1640, 10% Heat inactivated FBS, 1% Penicillin/Glutamine, 1% L-glutamine). The media was removed and replaced by epithelial strip buffer (PBS, 5 mM EDTA, 1 mM DTT, 5% Heat inactivated FBS, 1% Penicillin/streptavidin) and placed for 10 min in a 37°C water bath. Samples were strained using a 70μm strainer (Sigma-Aldrich) then centrifuged and re-suspended in RPMI media. This method generates cell fractions including both intraepithelial cells and lamina propria cells. For TCR sequencing, tissue cells were stored in RNAlater (Sigma-Aldrich) until when needed for DNA extraction.

#### DNA Extraction

DNA was extracted from stored tissue in RNAlater and PBMCs (Whole blood) using DNeasy Blood and Tissue kit (QIAGEN) according to the manufacturer's instructions.

### Flow Cytometry

PBMCs were thawed in RPMI media (RPMI 1640, 10% Heat inactivated FBS, 1% Penicillin/Glutamine, 1% L-glutamine) supplemented with DNase, washed twice by centrifugation (2,000 rpm for 5 min) and rested for 2 h at 37°C, 5% CO_2_, followed by centrifugation and resuspension of the pellet in 50 μl antibody mixture for 20 min at room temperature. Cells were washed twice (PBS) and fixed with 2% PFA. Cells were then acquired using Aria Fusion III cytometer (BD) and analyzed with FlowJo software, version 10 (Tree Star). Cells collected from tissue were stained with same antibody mix consisting of: LIVE/DEAD Fixable Near-IR Dead Cell Marker (Invitrogen, Thermo Fisher Scientific); anti-CD45 V500, clone Hi30; anti-CD4 Brilliant Violet 496, clone SK3; anti-CD8 Brilliant Violet 395, clone RPA-T8 (All from BD); anti-CD3 Brilliant Violet 785, clone OKT3; anti- Vα7.2 Brilliant Violet 711, clone 3C10; anti-CD161 BV605, clone HP3G10; anti-CD26 PE-Cy5, clone BA5b; anti- γδ TCR PE, clone B1; anti-Vγ9 TCR APC, clone B3; anti-Vδ2 TCR PerCPCy5.5, clone B6 (All from BioLegend); anti-Vδ1 TCR FITC, clone TS8.2 (Thermo Fisher Scientific).

### Mass Cytometry (CyTOF)

An adapted version of a published protocol (33) was performed as follows: Fresh tissue cells were counted and stained in 100 μl containing APC-CD45 (1:20) and incubated on ice for 20 min. Hundred microliter of cold FACS buffer was added and centrifuged for 1,500 rpm for 3 min at 4°C, the supernatant was discarded, and the pellet resuspended in 100 μl cold cyFACS buffer. PBMCs were counted in 1 ml of cold cyFACS buffer and a maximum of 3 × 10^6^ live cells resuspended in 100 μl of cold cyFACS buffer. Both tissue cells and PBMC were kept on ice throughout the staining process.

A calculated amount of cyFACS buffer was added into 0.1 μm Ultrafree® centrifugal filter unit followed by appropriate volume of each antibody into the filter unit. The antibody cocktail was centrifuged at 14,000 rpm for 3 min at 4°C and the flow through antibody cocktail used to stain the cells. Hundred microliter APC-CD45 positive tissue cells was mixed with 100 μl of matched PBMC. The total number of cells stained varied between 3 and 6 × 10^6^. The mixed samples were then centrifuged at 1,500 rpm for 3 min at 4°C and cells resuspended in 100 μl of freshly diluted cisplatin (1:1500 in cyFACS buffer) and incubated on ice for 5 min. Hundred microliter of cyFACS was added to the cells to quench the reaction then centrifuged at 1,500 rpm for 3 min at 4°C. Cells were resuspended in 50 μl of cyFACS buffer containing anti-human CD294 CRTH2 (diluted 1:10) and anti-human γδ TCR (dilute 1:25) primary antibody then incubated at 37°C for 15 min followed by two washing steps in cyFACS at 1,500 rpm for 3 min at 4°C. Cells were resuspended in 50 μl of antibody cocktail panel solution, incubated on ice for 30 min. Cells were washed twice in cyFACS buffer then once with PBS and resuspended in 150 μl of freshy reconstituted 2% PFA. The plate was sealed with an adhesive sealing film and stored overnight at 4°C. The cells were centrifuged at 1800 rpm for 3 min at 4°C, supernatant discarded followed by one more wash in 200 μl of cyFACS buffer. The cells were resuspended in 150 μl of cold freezing media and stored at −80°C until later for CyTOF acquisition.

After thawing, cells were washed in 1X permeabilization buffer and each sample was incubated with 50 μl of intracellular antibody in 1X permeabilization buffer for 30 min on ice. Cells were washed twice, and each sample was barcoded with a unique combination of two distinct palladium barcodes for 30 min on ice. After washing, cells were resuspended in 250 nM iridium intercalator (DNA staining) in 2% paraformaldehyde/PBS at RT. Cells were washed and adjusted to 0.5 million cells per ml H_2_O together with 1% equilibration beads (EQ Four element calibration beads, Fluidigm) for acquisition on a CyTOF® Helios system.

Signals for each parameter were normalized based on EQ beads added to each sample. Any zero values were randomized using a custom Rscript that uniformly distributes values between minus-one and zero. Each sample was manually de-barcoded followed by gating on DNA^+^ cells. Immune cells were identified by gating on live (cisplatin-) CD45^+^ cells and tissue and PBMCs from the same donor were further identified according to the tissue-specific live cell barcode tag (APC-CD45). Subset identification followed a gating cascade according to the lineage markers using FlowJo (Tree Star Inc) software. High dimensional data analysis was performed using immunoSCAPE's cloud-based analytical pipeline tool Cytographer®. For the visualization of high dimensionality data, Uniform Manifold Approximation and Projection (UMAP) as dimensionality reduction technique was used. Phenotypic dissection was performed using manual and usupervised clustering (PhenoGraph). Marker expression intensities were represented as heatmaps and expression plots.

### TCR Sequencing

For TCRα sequencing, purified genomic DNA was sequenced by Adaptive Biotechnologies using the ImmunoSEQ assay (http://www.immunoseq.com) as described (34). PBMCs were subjected to deep-resolution sequencing (identified TCRs with a frequency of 1 in 2 × 10^5^ to 1 × 10^6^), and gut tissue samples were subjected to survey resolution (1 in 60,000), we reasoned that survey-level sequencing was sufficient for identification of expanded TCRs in the gut, but a greater in-depth might be required identify those TCRs if they were less expanded in the blood (31). Data were analyzed using the ImmunoSEQ analyser tools. Productive rearrangements (in-frame without stop codons) and TCR gene segment assignment were done as part of the ImmunoSEQ assay. The list of final clones generated by ImmunoSEQ was analyzed using VDJ tools (version 1.2.1) (35). Throughout our analysis, we applied the following filters: frame = in and reads >1. The filter applied for TCR analysis of unconventional T-cells is summarized in **Figure 5B**. In most of our analyses we stated TCR frequencies as a fraction of 1. MAIT Match server (http://www.cbs.dtu.dk/services/MAIT_Match/) was used to identify additional TCRs with MAIT-like features (36). TCR-α repertoire normalized data has been supplied in Supplementary Tables 2–4.

### Statistical Analyses

Data are presented as the mean ±SEM. A 2-tailed Student's *t*-test was used for normally distributed data for comparison of two groups. 2-way ANOVA was used for comparison of more than two groups, followed by Dunn's or Sidak's post-test. A *p* < 0.05 was considered statistically significant. Statistical and graphical analyses were performed using GraphPad Prism, version 8.4 (GraphPad software).

## Results

### Distinct Phenotype Clustering Supports Tissue-Specific Compartmentalization of Unconventional T-Cells Subsets

**Figure 1:**
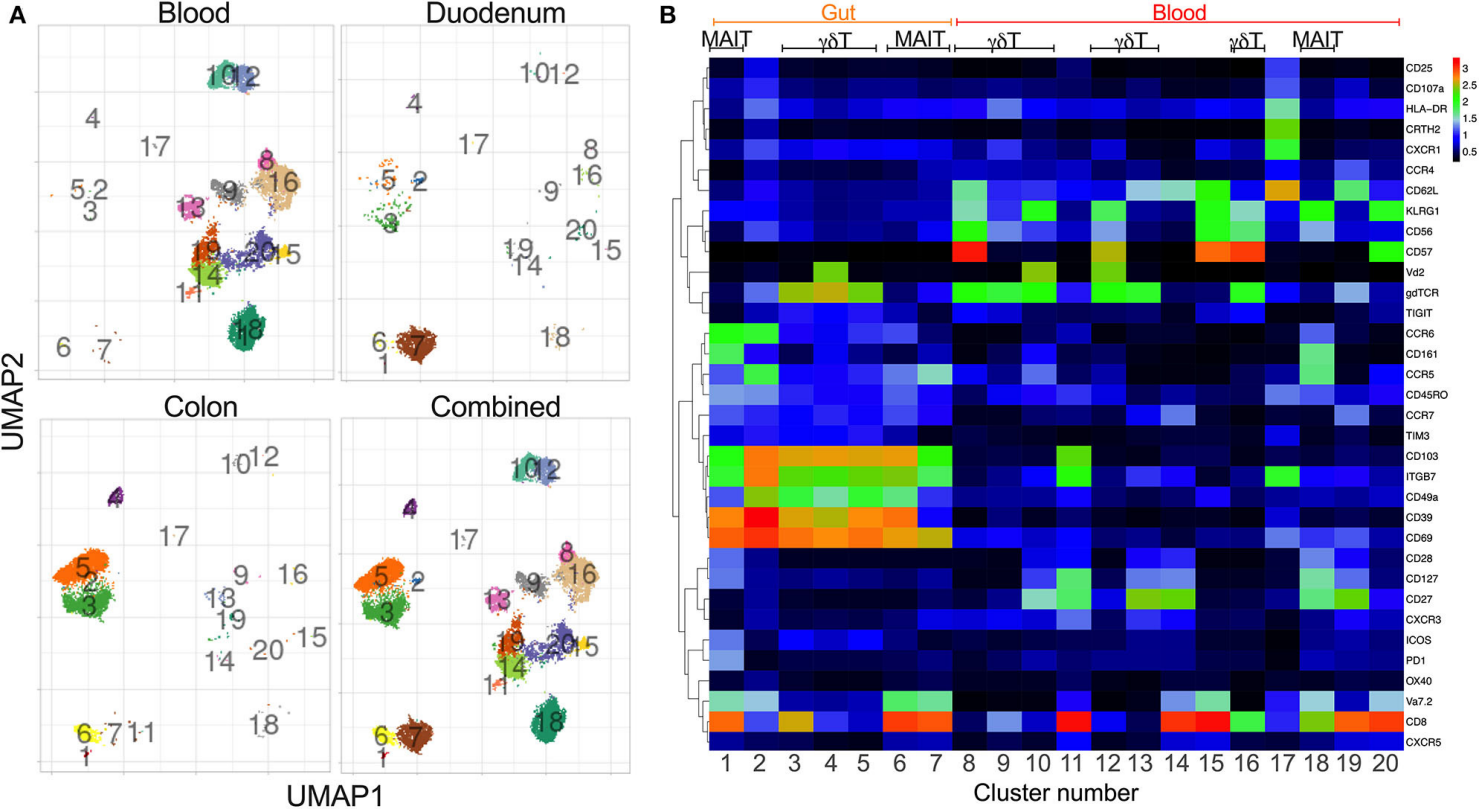
Distinct phenotype clustering supports tissue-specific compartmentalization of unconventional T-cell subsets. Mass cytometry was performed on three colon (022-09-1140, 022-09-1182, 022-09-1183) and four duodenum samples (022-09-3120, 022-09-3121, 022-09-2187, 022-09-2188) with matched blood (see Supplementary Table 1 for individual participants' characteristics). All participants were HIV negative. **(A)** Uniform manifold approximation and projection (UMAP) of blood, colon and duodenum of unconventional T-cell subsets (CD3^+^Vα7-2^+^γδTCR^+^; gating strategy Supplementary Figure 1A). Events are color-coded according to cell marker clusters. **(B)** Heatmap shows the frequency of surface marker expression within the manually gated clusters from **(A)**. Scale bar indicates Log 2 of positive expression from low (black) to high (red) of the indicated markers.

To generate a comprehensive overview of the phenotype and distribution of unconventional T-cells in the human gut, we first performed high dimensional cytometry by time of flight (CyTOF) analyses of matched blood and gut (duodenum, *n* = 4; colon, *n* = 3) samples of HIV uninfected individuals (Table 1) (Supplementary Figure 1A) using a panel of 41 antibodies. We identified 20 major populations of T-cells, differentially distributed between the 3 compartments studied, with the highest variety of cell subsets detectable in blood, followed by the colon and the duodenum (Figure 1A) (Supplementary Figures 1B,C, 2). As expected, markers associated with an earlier differentiation stage [CD62L, CD27; (37, 38)], were detectable in circulation (clusters 8–20) but absent in gut tissue samples (clusters 1–7). KLRG1, a marker associated with senescence and recent activation on NK and T-cells (39), was also exclusively detected in peripheral blood clusters, consistent with studies showing this marker is absent on tissue resident T-cells (40). As described previously, MAIT cells, identified by co-expression of CD161 and Vα7.2 (10) were present at high frequency in blood (cluster 18). Potential MAIT clusters were also observed in the colon (cluster 6) and in the duodenum (cluster 7), with MAIT-like cells in the colon uniquely expressing high levels of the integrin CD49a and CD39, a surface enzyme associated with regulatory function (41, 42). These clusters have lower expression of CD161 than in the blood, but are associated with an additional, smaller cluster in the UMAP projection, cluster 1, which does expresses high levels of CD161. Several γδ T-cell clusters are observed in the blood, including pronounced Vδ2^+^ population (cluster 10+12; Figure 1B). Again, distinct compartmentalization of γδ T-cells was observed in the gut, with all 3 γδ populations (cluster 3–5) more pronounced in the colon than in the duodenum. Interestingly, cluster 3, which lacks Vδ2, appears to be a CD8 T-cell population. This is in contrast to the blood, in which γδ T-cells do not express high levels of CD8, consistent with published data showing that ~70% of γδ T-cell in blood are CD4 and CD8 double negative (43). Overall, these data suggest clear compartmentalization of unconventional T-cells between the small and large intestine with MAIT cells dominant within the duodenum and γδ T-cells within the colon.

### Frequency of Unconventional T-Cells Is Associated With Concomitant Diseases in Peripheral Blood and Tissue

**Figure 2:**
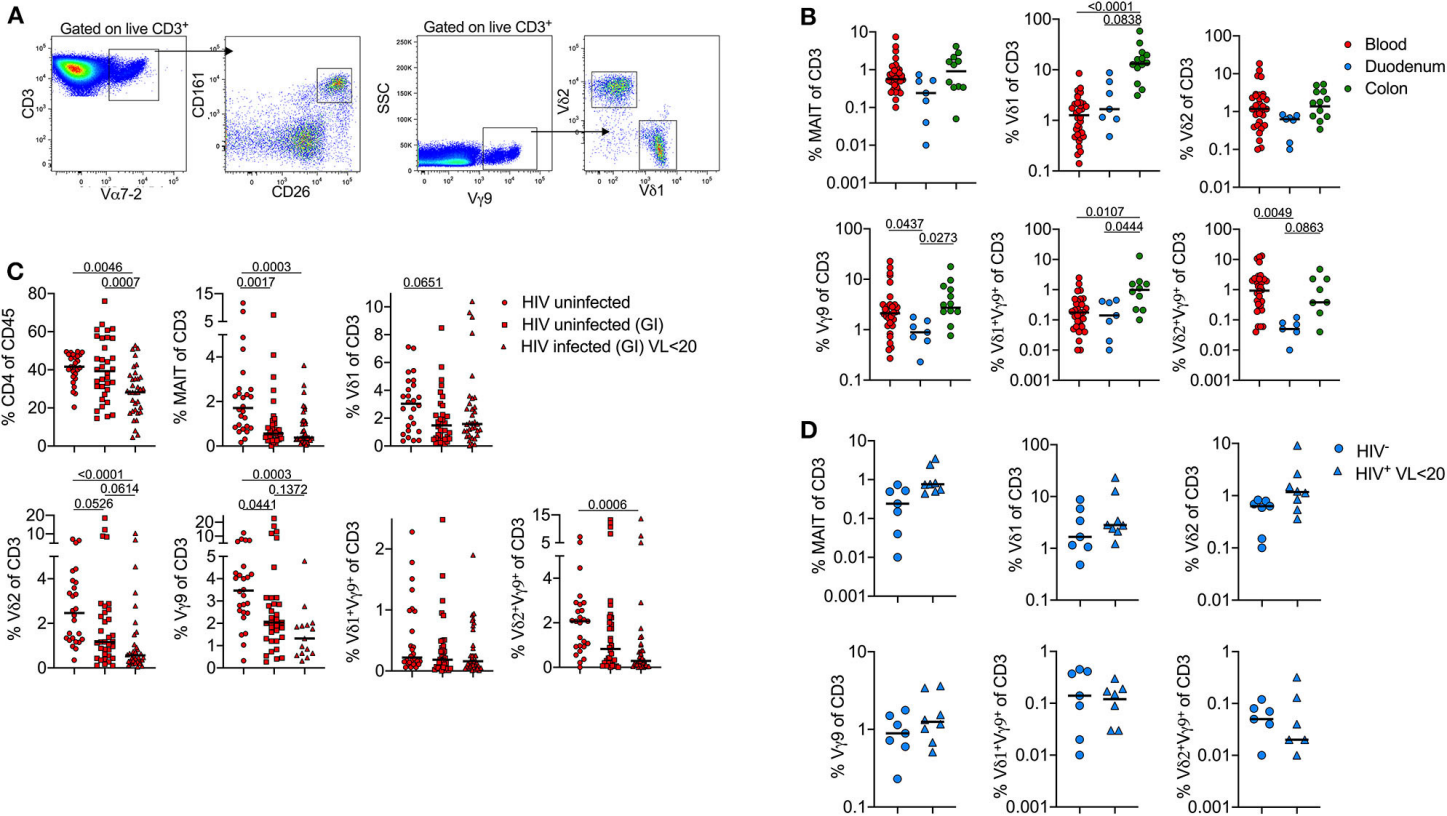
Frequency of unconventional T-cells is associated with concomitant diseases in peripheral blood and tissue-specific compartmentalization. Flow cytometry staining was performed on 75 participants (Table 1 and Supplementary Table 1), each dot represents an individual. **(A)** Gating strategy to identify donor-unrestricted T-cells: CD3^+^Vα7-2^+^CD26^+^CD161^+^ (MAIT cells); CD3^+^Vγ9^+^Vδ1^+^ and CD3+Vγ9^+^Vδ2^+^ T-cells. **(B)** Frequency (% of CD3 T-cells) of MAIT and γδ T-cells subsets in blood (red; *n* = 26), duodenum (blue; *n* = 7) and colon (green; *n* = 12). Only HIV-negative samples from GI cohort were included. **(C)** Frequency (% of CD3 T-cells) of MAIT and γδ T-cells subsets in blood; comparison between HIV –ve samples from FRESH cohort (circles; *n* = 26), HIV –ve (squares; *n* = 35) and HIV+ve samples (triangles; *n* = 21) from GI cohort. **(D)** MAIT and γδ T-cell subset frequencies in duodenum pinches; comparison between HIV –ve samples (circles, *n* = 7) and HIV+ve (triangles, *n* = 8) samples from GI cohort. Statistical analyses were performed by ANOVA and Dunn's multiple comparisons test in **(B,C)** and Mann-Whitney U test in **(D)**. Black lines represent median.

To test these observations in a larger HIV -ve cohort, we next used flow cytometry to quantify MAIT cells [Vα7.2^+^(TRAV1-02)CD161^+^CD26^+^] and γδT-cells (Vγ9 TCR antibody and Vδ1 or Vδ2) (Figure 2A). Although MAIT cells tended to be more frequent in the colon, this difference was not significant. However, we confirm that certain γδT-cells are highly enriched in the colon compared to both duodenum and matched blood samples (Figure 2B). Vγ9Vδ1^+^ T-cells in particular were highly expanded in the colon, but all γδT-cell populations were higher in this compartment than in the duodenum, and present at either similar or higher frequency than in the blood. By contrast, γδ T-cells tended to be lower in the duodenum than matched blood, particularly in the case of the Vγ9Vδ2^+^ subset.

Next, we examined the impact of HIV infection on MAIT cells and γδ T-cells. In blood, the frequency of MAIT cells was highly depleted in the blood of both HIV+ and HIV- participants from the GI clinic compared to non-GI HIV- healthy controls (Figure 2C). For HIV infected individuals this is consistent with multiple reports showing HIV infection leads to the loss of circulating MAIT cells (22, 24). However, the low frequency of MAIT cells in uninfected participants was unexpected and is likely related to ongoing gastrointestinal complications in these individuals. Likewise, the frequency of δ1, δ2 and Vγ9Vδ2^+^ T-cells is lower in both groups compared to healthy controls. However, δ2 and Vγ9Vδ2^+^ T-cells are especially depleted on HIV infected individuals, as reported elsewhere (28). By contrast, from the limited samples available, HIV infection did not lead to significant changes in frequencies of MAIT cells or γδT-cell subsets in the duodenum (Figure 2D). In contrast to the blood compartment, these data suggest that HIV infection does not appear to exacerbate depletion of unconventional T-cells in the gut, at least when identified by surface marker expression.

### Expansion of T-Cell Clones in Duodenum

**Figure 3:**
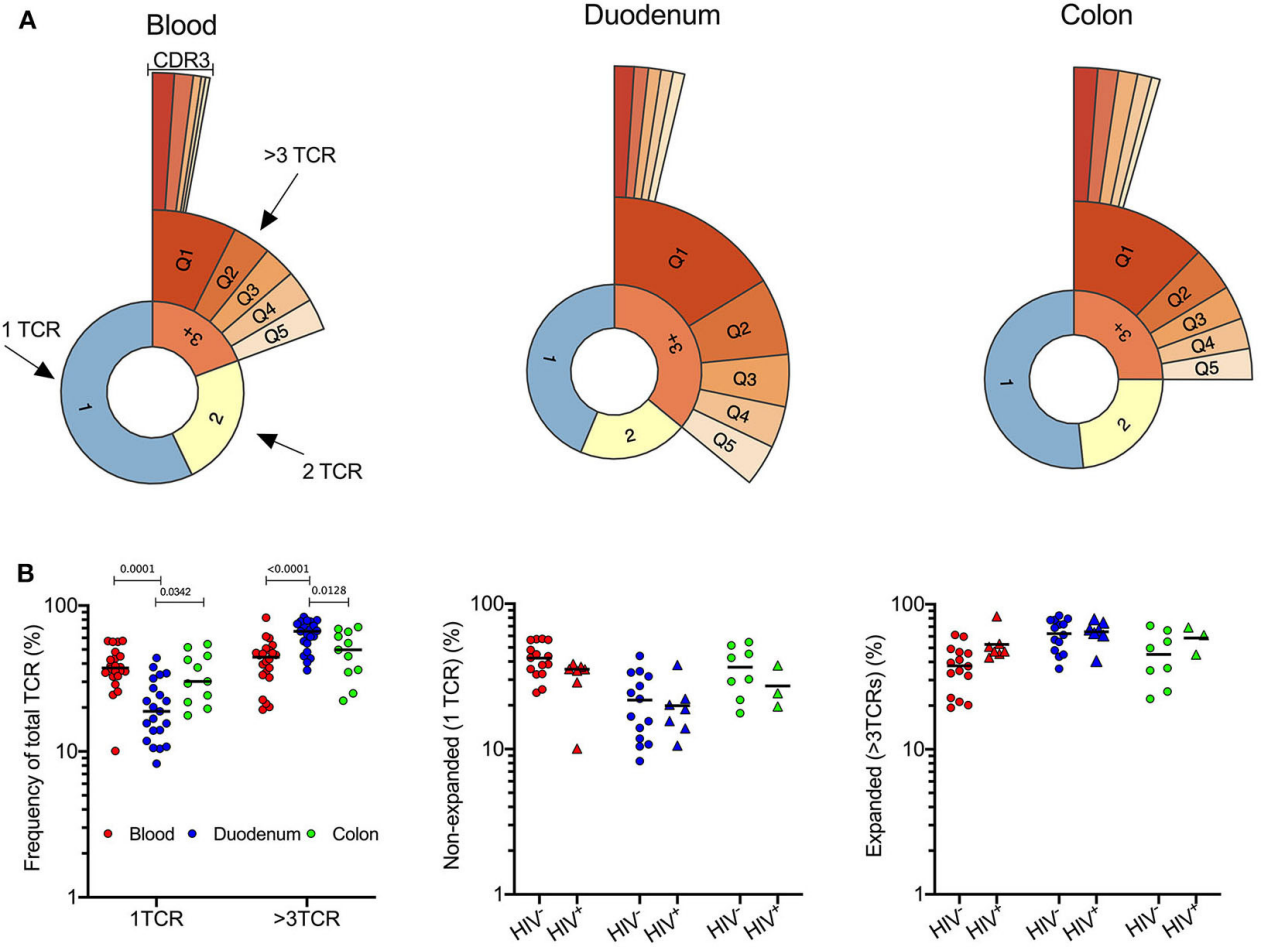
Expansion of T-cell clones in duodenal GALT tissue. **(A)** Quantile plot TCRα CDR3 clonality in 1 representative sample of blood, duodenum and colon (PID 022-09-1069, 022-09-2078, and 022-09-1070 respectively; Supplementary Table 1). All participants were HIV –ve. **(B)** Left: Frequency of singular CDR3 vs. >3 identical CDR3 of total TCRs sequenced as a measure of clonality in blood (red; *n* = 22), duodenum (blue; *n* = 21), and colon (green; *n* = 11) pinches. Plot includes HIV– and HIV+ participants. Frequency of singular (middle) and expanded (right) CDR3 sequences in blood (red; *n* = 22), duodenum (blue; *n* = 21), and colon (green; *n* = 11) pinches of HIV –ve (circles; *n* = 37) vs. HIV+ve (triangles; *n* = 17) individuals. Dunn's multiple comparisons test. Black lines represent median.

In light of this unexpected finding we turned to global TCR sequencing as an unbiased way of quantifying conventional T-cells, DURT and γδT-cells (31) within the colon, duodenum and matched blood compartments. First, to address the overall compartment-specific differences in the conventional T-cell repertoire, we excluded DURT and δ-TCR sequences and grouped TCRs according to whether each individual TCR occurred once, twice or more than 3 times within a sample (Figure 3A). Single TCRs were observed more frequently in the blood than in gut intestinal tissue, consistent with a higher frequency of naïve T-cells (Figure 3B). In contrast, the duodenum contained significantly more expanded TCRs (i.e., represented more than 3 times; Figure 3B), consistent with a predominance of clonally expanded memory T-cells in this compartment. Surprisingly, the same clonal expansions were not observed in the colon, suggesting conventional T-cells are typically more active in the duodenal mucosal than in the colon. When grouping participants according to HIV infection status, no significant differences in TCR clonal expansion could be seen in the compartments studied within our cohort of limited sample size (Figure 3B). As described previously (44, 45), there was a trend toward expansion of TCRs in peripheral blood, although not reaching significance with this sample size (Figure 3B).

### Compartment-Specific Expansion of γδ T-Cells in Colon and iNKT Cells in Duodenum

**Figure 4:**
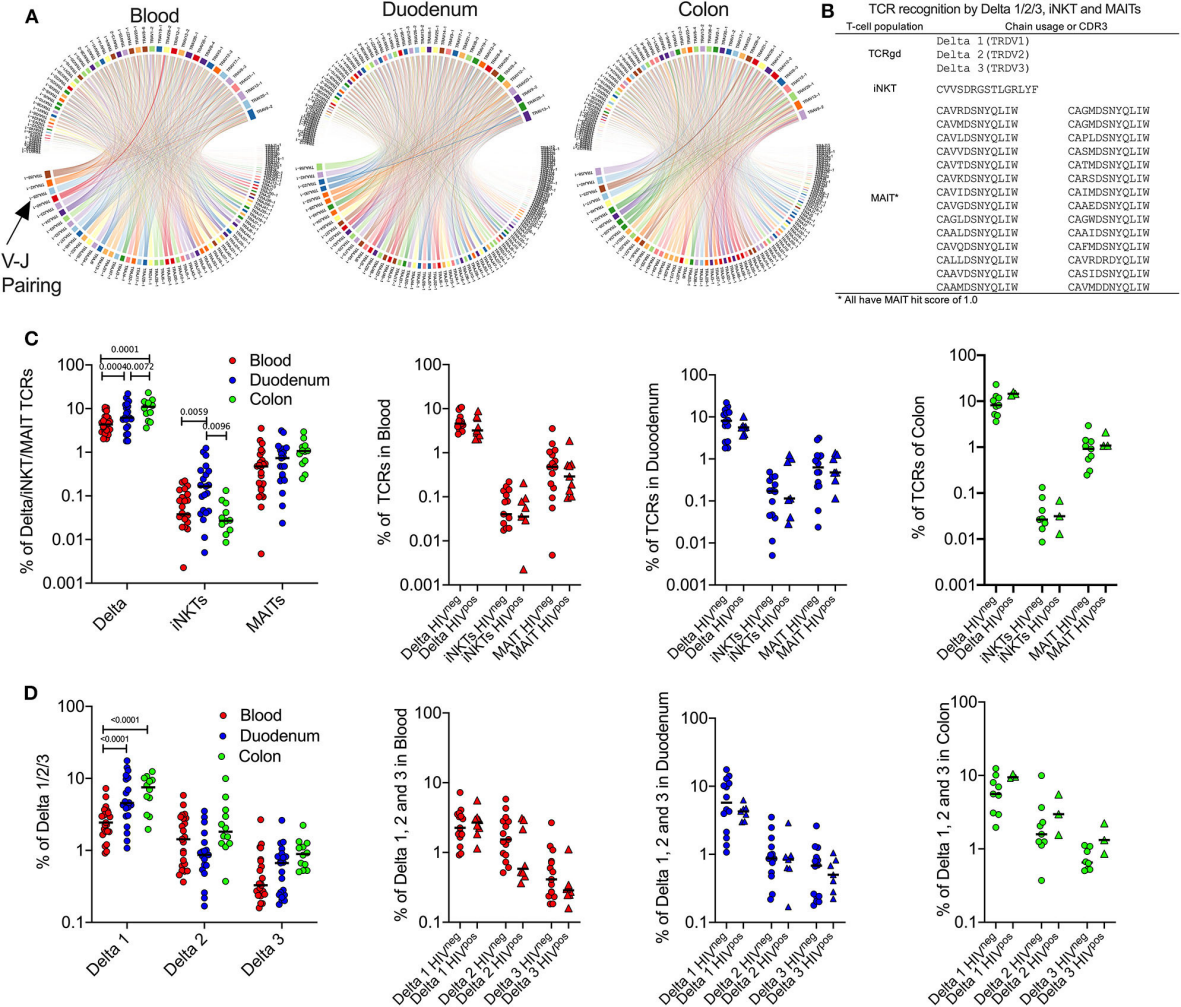
Compartment-specific expansion of γδ T-cells in colon and iNKT cells in duodenum GALT tissue. **(A)** TRAV-TRAJ segment usage wheels are shown for 1 representative sample of blood, duodenum and colon (PID 022-09-1069, 022-09-2078, and 022-09-1070, respectively; Supplementary Table 1). The size of the arcs is proportional to V or J frequency. The ribbon joining any V-J pair is proportional to the co-occurrence frequency of that pair. Co-occurrence (“circos”) wheels were generated by VDJ tools (35). All 3 participants were HIV –ve. **(B)** Overview of TCR sequencing-based definition of unconventional T-cell subsets used in this study. **(C)** Left: Frequency of unconventional T-cell subsets as defined by TCR sequences displayed in **(B)** in blood (red; *n* = 24), duodenum (blue; *n* = 21) and colon (green; *n* = 12) samples. Plot includes HIV– and HIV+ participants. Right panels: Same as in the left panel but comparing HIV –ve (circles) vs. HIV+ve (triangles) samples. **(D)** Same as in **(C)** but comparing frequencies of γδ T-cell subsets. Black lines represent median. Dunn's multiple comparisons test.

We next investigated differences in unconventional TCR repertoire of the blood, duodenum and colon as shown in representative HIV-uninfected samples (Figure 4A). We defined unconventional T-cells as γδ T-cells, iNKT cells and MAIT cells by usage of specific TCR-alpha or delta sequences listed in Figure 4B. As the antigens that MR1-restricted MAIT cells recognize are still being defined, the definition of MAIT TCRs is continuously changing. Therefore, we extended our definition of MAIT cells to include TCRs that scored 1.0 using an algorithm called “MAIT match,” which scores TCRs according to similarity to known MAIT cells sequences (36, 46). Using these definitions, we compared frequencies of unconventional TCRs between peripheral blood, duodenum and colon tissue compartments. Consistent with the cytometry data, the frequency of δ-TCRs was significantly increased in colon when compared to duodenum and blood samples, particularly δ-1 (Figures 4C,D). Conversely, iNKT cells, which were the least frequent subset of unconventional T-cells detectable, displayed a significant compartment-specific enrichment in duodenum when compared to blood and colon samples (Figure 4C) (11). As suggested by the flow cytometry data, MAIT TCRs tended to be more abundant in the colon than the duodenum, although this did not reach significance. In addition, we did not observe any impact from HIV infection on absolute frequencies of γδ T-cells, iNKT cells and MAIT TCRs within the compartments studied (Figures 4C,D), in contrast to previous published work (11, 26, 27). However, this could be explained by the limited sample size. Although δ1 T-cells were the main driver of overall γδ T-cell compartment specific changes, we found no impact from HIV infection in any of the three γδ T-cell populations. These data are consistent with our findings in Figure 2 and confirm that tissue compartment rather than a concomitant HIV infection has a dominant influence on the frequency of unconventional T-cell subsets in the gut.

### High Reproducibility of TCR Sequences Between Singular Duodenal Pinch Biopsies

**Figure 5:**
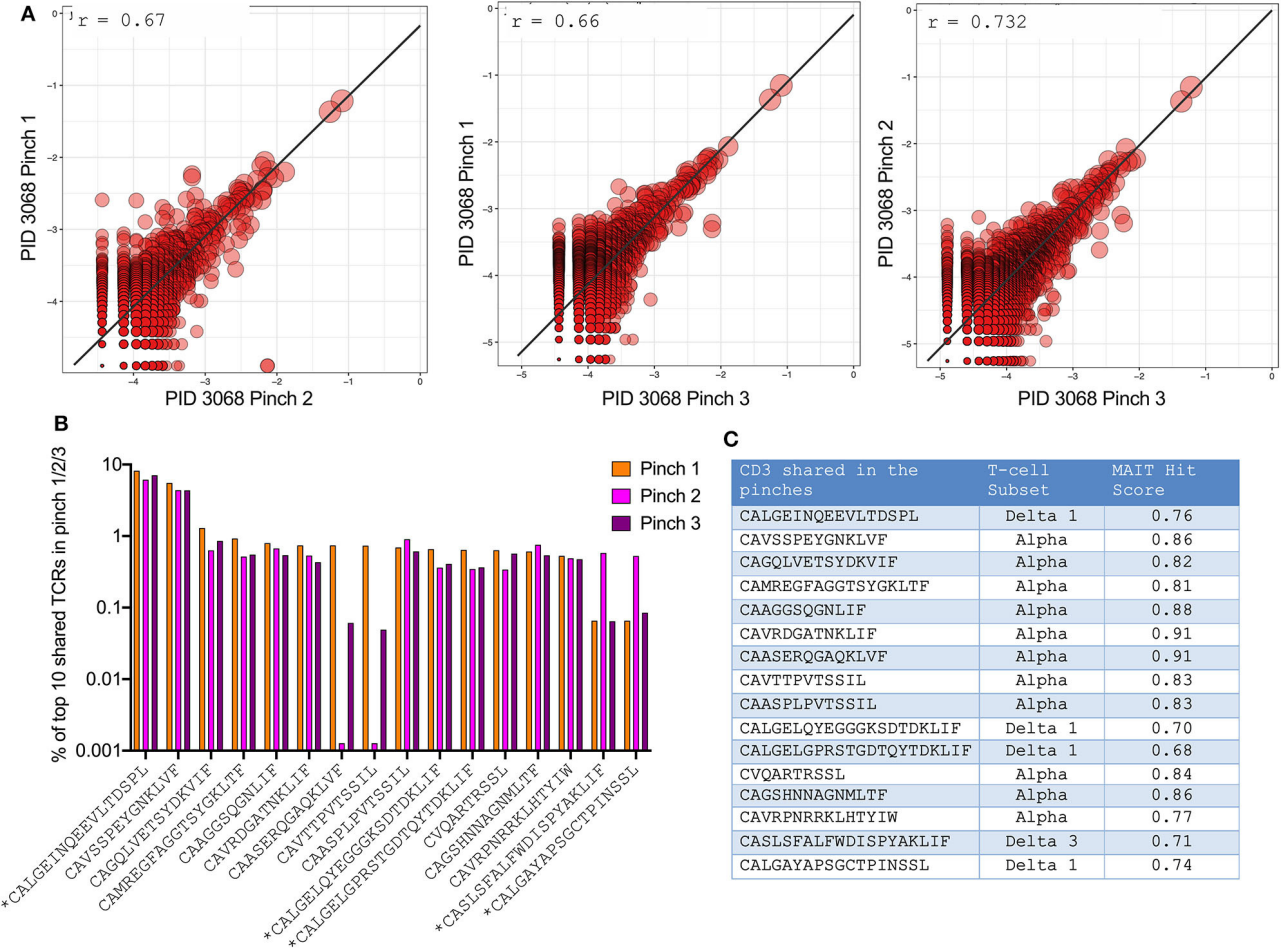
High reproducibility of TCR sequences between singular duodenal pinch biopsies. **(A)** Pearson correlation of TCR sequences obtained from three duodenal pinches of PID 022-09-3068 (participant is HIV –ve; Supplementary Table 1). **(B)** Frequency of the most abundant TCR sequences shared between the three pinches with * denoting δ (delta) T-cell subset. **(C)** Corresponding T-cell subsets to obtained CDR3 sequences that were shared between the 3 duodenal pinches as illustrated in **(B)**, MAIT hit score from (http://www.cbs.dtu.dk/services/MAIT_Match/) was used to identify additional TCRs with MAIT-like features.

Having observed significant differences in frequencies of unconventional T-cells by flow and TCR sequencing, we wanted to confirm the validity and reproducibility of singular pinch biopsy sampling within the small intestine. For this we compared TCR data in one individual from whom we were able to obtain 3 different duodenal biopsies. This revealed a highly significant correlation between TCRs identified within the 3 different pinches obtained (*r* = 0.66–0.73) (Figure 5A). In addition, the 10 most frequent TCR sequences in each pinch were the same in all 3 pinches, although some fluctuation in frequency was observed (Figure 5B). Notably, the most frequent TCR corresponded to either Vδ1^+^ T-cells or cells with an elevated MAIT-match score (46) (Figure 5C), confirming the dominant role of unconventional T-cells in this compartment.

### TCR Sharing of MAIT and iNKT, but Not γδ T-Cell Subsets Between Different Human Tissue Compartments

**Figure 6:**
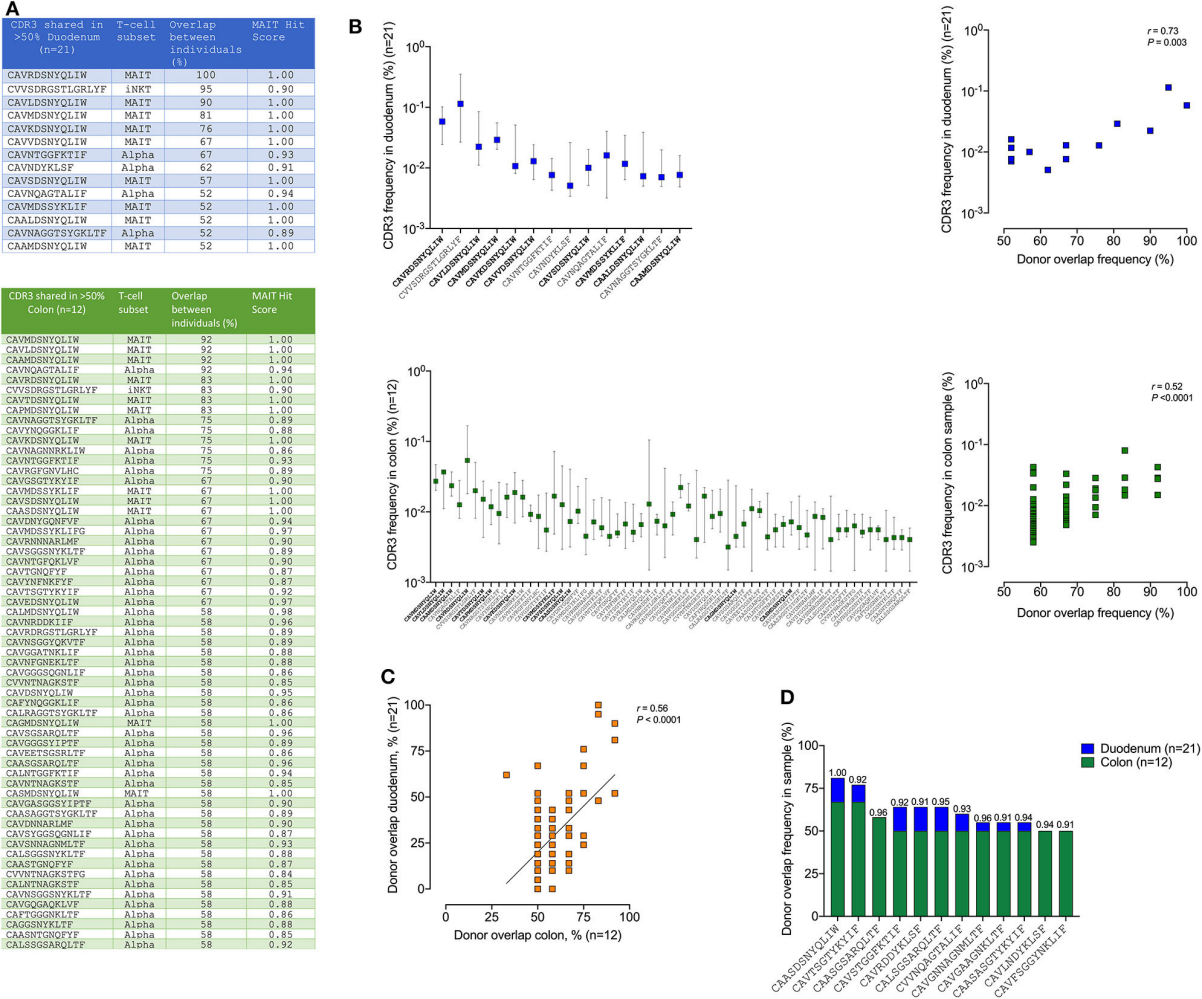
TCR sharing of MAIT and iNKT but not γδ T-cell subsets between different human colon and duodenum tissue compartments. **(A)** Shared CDR3 amino acid sequences present in more than 50% samples of duodenum (top panel blue, *n* = 21) and colon (bottom green, *n* = 12) with corresponding T-cell subsets for each CDR3 sequence displayed in the second column, the percentage overlap between participants and MAIT Hit Score was used to identify additional TCRs with MAIT-like features (http://www.cbs.dtu.dk/services/MAIT_Match/). **(B)** Median frequency of these shared CDR3s in duodenum and colon and correlation between the donor overlap frequency and the corresponding CDR3 frequency in each sample (right panel blue/green, *n* = 21/12), respectively with bold sequences on x-axis denoting MAIT cells. **(C)** Same data as in **(A)** but displayed as correlation between colon (green panel, *n* = 12) and duodenum (blue panel, *n* = 21) donor overlap with all CDR3s with minimum 50% donor overlap in either duodenum or colon shown. **(D)** CDR3s with MAIT hit score >0.9 significantly (Fisher's exact test *p* < 0.05) enriched in colon (*n* = 12) compared to duodenm (*n* = 21), with MAIT hit score shown above each bar (see Supplementary Table 5 for full table irrespective of MAIT hit scores).

Finally, to examine the regional tissue-specific distribution of unconventional T-cells and to detect potential novel DURT TCRs, we focused on TCRs shared between at least 50% of participants and compared the overlap of TCR alpha sequences between participants within the duodenum and the colon (Figure 6A). This is based on the assumption that, in genetically diverse individuals, only DURTs are likely to be highly shared (47). Interestingly, based on this criteria, we found far more TCR overlap within the colon compared to duodenum (>50%, colo*n* = 61, duodenum = 14), with these sequences comprised of either known MAIT TCRs, TCRs with a high MAIT score (46) or iNKT cells. No shared delta chains were detected, and we find no clear evidence of novel DURT sequences (i.e., shared TCRs with a low MAIT score). We then ranked the TCRs by their percent overlap between donors and plotted their overall median frequency. This showed a direct positive correlation within both tissue compartments (Figure 6B), and between compartments (Figure 6C), suggesting a consistent and expected general relationship between TCR sharing and abundance. However, when comparing shared repertoires within the colon vs. the duodenum we found that 45 of the TCRs were significantly more shared in colon than in duodenum (Supplementary Table 5), of which 12 had strong “MAIT-like” CDR3 sequence (MAIT hit score >0.9) and one was a canonical MAIT TCR (score 1.00) (CAASDSNYQLIW) (Figure 6D). Of note, three of these TCR alpha CDR3 sequences were present in 50% or more of the 12 participants in colon, but found in none of 21 duodenum samples analyzed (*p* < 0.001). This data, therefore, strongly supports the existence of compartmentalization of the “MAIT-like” DURT TCR repertoire within the gastrointestinal tract.

## Discussion

The distribution of unconventional T-cells, including MAIT cells, iNKT cells and γδ T-cells, within human gastrointestinal tract, and the impact of HIV on these subsets, has not been well-established. In this study we used high dimensional mass cytometry, conventional flow cytometry and unbiased global TCR sequencing to study the distribution of these important immune cells in the small and large intestine, and evaluated the impact of HIV infection. These combined approaches confirmed the existence of compartmentalization of unconventional T-cells between intestinal sites. However, with this sample size, we find no evidence that treated HIV infection induces significant skewing of these T-cell subsets within intestinal tissue.

Overall, we find an enrichment of iNKT cells and clonally expanded conventional TCRs in the duodenum, whilst γδ T-cells, particularly those expressing the δ-1 chain, are preferentially expanded in the colon. Moreover, there is a trend for more abundant MAIT cells in the colon, and TCR sequencing supports the existence of a significantly more diverse MAIT-like population in this compartment compared to the duodenum. These differences are likely to reflect the distinct anatomical and physiological characteristics of the small and large intestine, including the microbial communities they support, which increase in size and complexity as the intestinal tract descends (30).

The increased frequency of iNKT cells in the duodenum is consistent with data from mice, in which iNKT cells are also more abundant in the small intestine than in the colon (48). This has been attributed to inhibitory effects of sphingolipids produced by the anaerobic bacteria *Bacteroides fragilis* within the colon, which limit iNKT expansion in this compartment during development (49). The clonal expansion of T-cells in the duodenum compared to matched blood is highly suggestive of the expansion of antigen-specific T-cells in this part of the intestinal tract. The fact that this is not observed in the colon suggests the adaptive immune response is more focused in the upper intestinal tract, which also represents the first line of defense against environmental pathogens (50). It may also be an important consideration in studies examining pathogen specific immunity in colon samples, as these data indicate they may be limited.

In contrast to conventional T-cells and iNKT cells, δ-1 expressing T-cells are clearly expanded in the colon compared to the duodenum. Human Vγ9δ1^+^ T-cells are mostly found in mucosal tissues and regarded important for intestinal barrier function, while Vγ9Vδ2^+^ T-cells predominate in the peripheral blood and secondary lymphoid organs (15). This shift from Vδ2 in the blood (and duodenum) to Vδ1 in the colon could be observed through flow cytometry and TCR sequencing. Unlike for DURTS, we find no evidence of shared TCRδ-sequences between individuals, or between tissue and blood. This is in line with previous work showing that that intestinal Vδ1^+^ T-cell repertoire was found to have no overlap with the blood and also strongly differed between the small and large intestine (51). However, within individual pinches, delta sequences were often highly expanded, and in the one individual from whom multiple samples were sequenced, the same Vδ1 clone (CALGEINQEEVLTDSPL) dominated all three pinches. This shows that γδ T-cells can undergo highly localized expansions as they encounter activating signals in response to microbes (14). In addition to δ1-TCRs, we also find evidence of an expanded MAIT repertoire in the colon. By both flow cytometry and TCR sequencing, which used samples from different participants, there is a trend for more abundant MAIT cells in this compartment. More strikingly, the MAIT repertoire appears to be far more complex in the colon than the duodenum, including 3 highly shared MAIT-like TCRs that are not seen in any of the 21 colon pinches sequenced. Although the precise relationships between MAIT and MAIT-like TCRs and their antigen specificity is yet to be established, MAIT ligands appear to be much more diverse than previously appreciated and are recognized by distinct MAIT TCR sequences (52). Therefore, these observations imply greater MAIT activity in the large intestine, which may relate to the more abundant and complex microbial communities found there. More work is required to unpick the mechanisms behind the compartmentalization of T-cells observed in the study, which may involve structural differences in addition to differences in the microbial communities and pathogen exposures associated with each site.

HIV infection has been shown to have a profound impact on unconventional T-cell frequency and function in peripheral blood and tissue (11, 22–25, 53). We observed in our flow cytometry experiments a depletion of MAIT and γδ T-cell populations in blood of virus suppressed HIV infected individuals in line with previous studies (22, 24, 28), but no difference within small and large intestinal tissue by either cytometry or TCR sequencing, in contrast to previous published data suggesting depletion of MAIT cells in the colon of HIV infected individuals (22). One caveat of this study is that it lack intestinal samples from healthy controls, as all individuals are undergoing medical examination for suspected gastrointestinal complications. It is possible that MAIT frequencies in the GI tract are reduced in all study participants compared to individuals not requiring medical investigation for GI complications. However, the frequency of MAITs detected by flow cytometry in healthy colon biopsies has been reported elsewhere as ~1% of CD8 alpha beta-T-cells (54), similar to the frequencies observed in this study by both flow cytometry and TCR sequencing. These data are also in line with Leeansyah et al. showing no MAIT cell depletion in the rectal mucosa despite reduction in the blood compartment of HIV infected individuals (24). It is important to note, however, that, based on data from the blood, the functionality of these cells is likely to be impaired (24, 54). Further studies with larger numbers of healthy participants (e.g., subjects consenting to research biopsies) will be needed to explore in more depth the impact of HIV infection on unconventional T-cells in the different tissue compartments and to investigate potential distinctions between the lamina propria and intraepithelial intestinal tissue compartments (55).

The data also raise the interesting observation that both MAIT and γδ T-cell populations in the blood are perturbed by common to many human conditions intestinal conditions in addition to widely reported associations with HIV and TB. This is illustrated by our observation that the frequencies of unconventional T-cells in peripheral blood were higher in the healthy controls (without reported gastrointestinal diseases) (32) than in the HIV negative GI-cohort. This is consistent with data showing that MAIT are highly depleted from the blood of individuals afflicted with Crohn's disease and ulcerative colitis (56). Interestingly, this is associated with an increase of frequency of MAITs with in the ileum. Likewise, γδ T-cell are reported to be significantly reduced in subjects with Crohn's disease (57).Although these conditions are not common in our study population, they do link gastrointestinal pathology with a loss of DURTs from the blood.

A unique feature of this study is its use of three different methodologies to observe similar evidence of compartment specific difference in the unconventional T-cell repertoire. In particular, we believe identification of T-cells using TCR sequencing removes any bias which can be caused by down regulation of some surface markers during activation associated with diseases such as HIV. Importantly, we have previously shown a strong correlation between both MAIT and δ-TCR frequency measured by sequencing and conventional flow cytometry (31). In addition, this approach readily identified differences in the MAIT TCR repertoire between the colon and duodenum that would not have otherwise been apparent. Taken together, these data provide important insight into the distinct unconventional TCR repertoires of the small and large intestine and how these differ from what can be observed in circulation.

## Data Availability Statement

The datasets presented in this study can be found in online repositories. The names of the repository/repositories and accession number(s) can be found in the article/Supplementary Material.

## Ethics Statement

The studies involving human participants were reviewed and approved by Biomedical Research Ethics Committee of the University of KwaZulu-Natal for all the studies. The patients/participants provided their written informed consent to participate in this study.

## Author Contributions

MM, AS, PO, and OA performed experimental work and data analysis. MF supported data analysis on Mass Cytometry. FK, TN, and FA provided clinical samples. JR contributed to manuscript writing and intellectual input. AL and HK wrote the paper and supervised the work. All authors contributed to the article and approved the submitted version.

## Conflict of Interest

MF is a shareholder and employee of immunoSCAPE Pte Ltd. The authors declare that the research was conducted in the absence of any commercial or financial relationships that could be construed as a potential conflict of interest.

## Supplementary Material

Supplementary Figure 1 **(A)** Gating strategy of blood, duodenum and colon gut tissue samples as lymphocytes/singlets/live/CD45^+^/CD3^+^ then gated for gdTCR/Va7.2 before unsupervised clustering using Cytographer, Immunoscape. **(B)** Relative frequency of each of the 21 clusters for colon, duodenum and blood and shown in **(C)** as stacked bars for each compartment and color-coded by cluster ID.

Supplementary Figure 2 Relative staining intensity of each marker in 36 markers used for sub-clustering using Cyographer software with all samples combined into one UMAP plot. Phenograph UMAP (last plot) identifies each cluster 1–20.

Supplementary Table 1 Clinical information of all individuals participants included in the study (*n* = 111).

Supplementary Table 2 TCRs found in blood (*n* = 33).

Supplementary Table 3 TCRs found in duodenum (*n* = 21).

Supplementary Table 4 TCRs found in colon (*n* = 12).

Supplementary Table 5 CDR3 sequences with 50% or more overlap between colon samples and their presence in duodenum samples shown as percent sharing (donor overlap).
