# Supplementary figures and images for: Unbiased Profiling Reveals Compartmentalization of Unconventional T-Cells Within the Intestinal Mucosa Irrespective of HIV Infection
Source: Front Immunol. 2020 Sep 30;11:579743. doi: 10.3389/fimmu.2020.579743 (PMC7561384; doi:10.3389/fimmu.2020.579743)

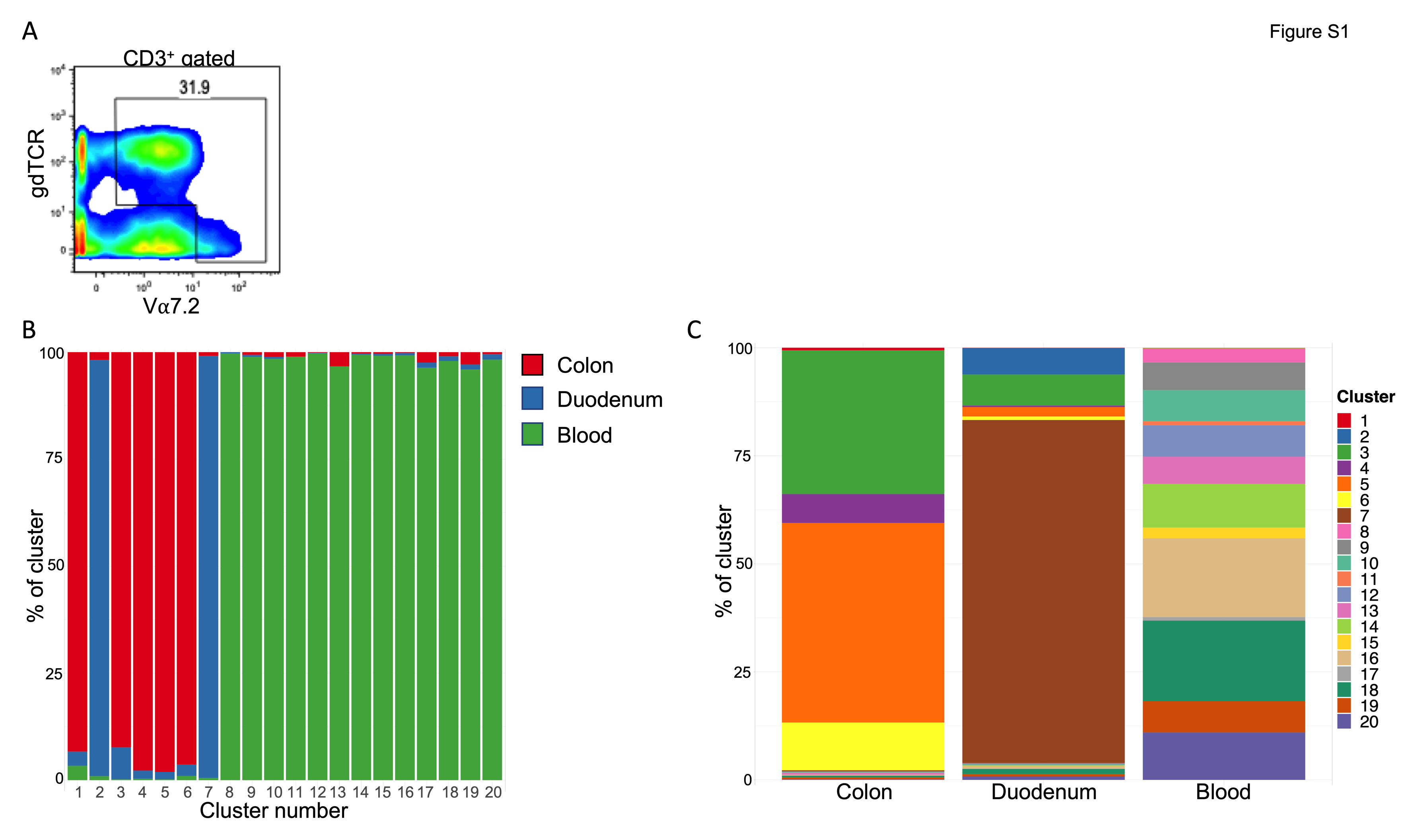

Supplement: Supplementary Figure 1 — (A) Gating strategy of blood, duodenum and colon gut tissue samples as lymphocytes/singlets/live/CD45+/CD3+ then gated for gdTCR/Va7.2 before unsupervised clustering using Cytographer, Immunoscape. (B) Relative frequency of each of the 21 clusters for colon, duodenum and blood and shown in (C) as stacked bars for each compartment and color-coded by cluster ID. [file Image_1.TIFF]

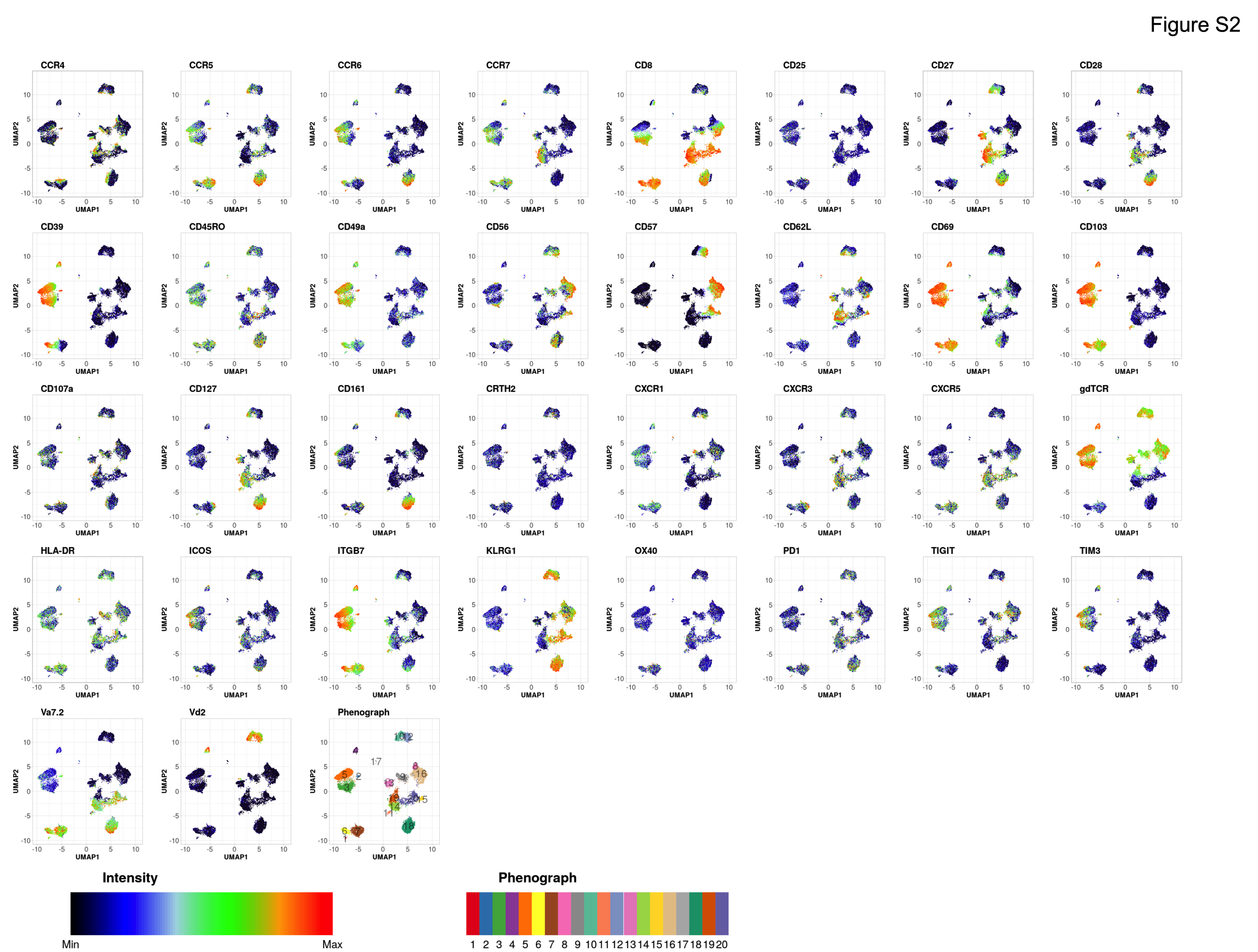

Supplement: Supplementary Figure 2 — Relative staining intensity of each marker in 36 markers used for sub-clustering using Cyographer software with all samples combined into one UMAP plot. Phenograph UMAP (last plot) identifies each cluster 1–20. [file Image_2.TIFF]
